# Supplementary material for: Learning curve and surgical outcome of robotic assisted colorectal surgery with ERAS program
Source: Sci Rep. 2022 Nov 29;12:20566. doi: 10.1038/s41598-022-24665-w (PMC9709162; doi:10.1038/s41598-022-24665-w)
Supplement: Supplementary file 2 — Supplementary Information 2. [file 41598_2022_24665_MOESM2_ESM.zip › RAL Raw data-Ñ[▒K/134/HS_ñw╝╨░O▒Kñσ.pdf]

正式摘要(電子簽章：000000232613220)<br>W92 / W92 ☐ 英文標題(注意:無英文姓名-請補病人基本檔!)	☐【讀取】憑證	

臺中榮民總醫院

出院病歷摘要

轉入號 : 轉出號 :

[redacted]

**(8)**流水編號	
**(9)**入院日期	110/12/15 (2021) 直腸外科 W92 - 09
**(10)**轉科日期	
**(11)**出院日期	110/12/19 (2021) 住院天數計 4 日

診斷

- 入院 :1. Sigmoid colon cancer
  2. DM, under regular medication
- 出院 :1. Sigmoid colon cancer, s/p Robotic assist Anterior Resection on 2021/12/16
  2. DM, under regular medication

主訴

- Colon cancer noticed during health exam

病史

- This is a 48 years old male patient with history of
  1. DM, under regular medication
  According to the patient, he had regular health exam at LinShin Hospital on 2021/11/16. Colonoscopy was done there, and sigmoid colon tumor with biopsy revealed adenocarcinoma. Abdominal CT was done there and showed no obvious distant metastasis. Thus, he came to our CRS OPD for second opinion. After discussion, this time he was admitted to our ward for surgival intervention.

體檢發現

-
  - General appearance :
    - easy looking , conscious clear , without cardiopulmonary distress
    - Vital signs自動帶入date 體溫(BT) 脈搏(P) 呼吸(R) 血壓(BP) 身高(BH) 體重(BW) BMI
      20211215 36.8℃ 97次/分 16次/分 166/119mmHg 168cm 68kg 24.1
    - Vital signs: B.P. 166/119 mmHg , P.R. 97 /min , R.R. 16 /min , B.T. 36.8 ℃
  - HEENT :
    - Eyes: no anemic conjunctiva, no icteric sclera, normal EOM
    - ENT: no otorrhea, no rhinorrhea, no erythematous mucosa, no injected tonsil, no deformity
  - Neck: supple, freely movable, trachea midline,no deformity, no engorged jugular vein , no palpable thyroid or lymph node enlargement
  - Chest:
    - Inspection: Grossly normal, symmetric and fully expansion, no accessory muscle used, no operation scar, no spider angiomata, no gynecomastia
    - Palpation: no palpable mass or nodule, no subcutaneous emphysema, no tenderness
    - Ausculation: clear breathing sound
  - Heart:
    - Palpation: no thrill, no heave
    - Percussion: normal heart size
    - Ausculation: regular heart beat, normal S1, S2, no gallop, no murmur
  - Extremities: No deformity, full ranged of motion without limitation, no clubbing finger or nail cyanosis, no pitting edema, no ulcer or pigmentation, no obvious skin lesion, no varicose vein, no bone, joint or muscle tenderness

  CRS CONDITION
  - Karnofsky performance status: (100) Normal, no complaints, no evidence of disease

-
  - PE of Abdomen:
  - CEA: . ng/ml
  - Imaging Studies:
  - Colonoscope report: .
  - Figures:
    1. colon & rectum

手術日期及方法

- 手術日期及方法20211216 1. Robotic (XI) assisted anterior resection
  2. Intraoperative colonoscopy : (-).
  3. High ligation (+) : At IMA root. Left colic artery and IMV were also ligated via robotic endoclips.
  4. Take down from the splenic flexure : (-)
  5. Robotic endoGIA (45-4.3) X2 + EEA (29mm) were applied
  6. Proximal cut end: 10 cm, distal cut end: 5 cm
  7. ADHESION BARRIER FILM was placed below the central abdominal wound

住院治療經過

- After admission, he received Robotic assist AR on 2021/12/16. He start try Bland I on 12/17, Bland II on 12/18. Under relative stable condition, he discharge on 2021/12/19, arrange OPD follow up.

合併症

一般檢查

- 累積報告 :

DATE	TIME	ALB	TP	BIL.T	BIL.D	ALKP	AST	ALT	LDH	AMY	LIPASE	NH3	Ferritin	UA
1101215	1948		7.6	0.73				51						7.3
1101216	1415	4.1		0.93	0.27	74	27	50						

DATE	TIME	WBC	RBC	HGB	HCT	MCV	PLT	BLST	NEUT	BAND	SEG	LYM	MON	EOS	BAS
1101215	1944	9550		14.0	42.3		235		63.3			27.4	4.6	4.1	0.6
1101216	1417	16410	4.64	13.0	38.4	82.8	222		90.3			5.7	3.7	0.1	0.2

DATE	TIME	NA	K	CL	BUN	CREAT	eGFR	CA	Mg	P	CRP	ESR	PCT	D-Dimer	LACTATE
1101215	1948	140	4.7		14	1.18	70.03				0.198				
1101216	1415	138	4.0		12	0.88	98.24	8.1							

DATE	TIME	GLUCOSE	HbA1C	CHOL	TG	HDL	LDL	KETONE	CK	CKMB	TRO-T	PT-P	APTT-P	NT-pro BNP
1101215	1948			201	82	39	138							

特殊檢查

病理報告

其它

出院時情況

- 15.計劃性出院

出院指示

-

藥名	劑量	劑量單位	用法	頻次	數量	數量單位	現況	天數	單位
(口)Takepron OD tab 30mg	1	TAB	PO	QDAC	10	TAB	TH	10	天
Mag. Oxide tab	2	TAB	PO	TID	60	TAB	TH	10	天
Ultracet tab	1	TAB	PO	Q6HPRN	20	TAB	TH	5	天

出院計劃

- 門診日期
  - date on 2021/12/29

主治醫師 2062F 蔣鋒帆 / 住院醫師 2339C 蔡濬樟

正式摘要(電子簽章：**000000232613220)**

W92 / W92

doctorRank:B
